# Effect of TLR4/MyD88/NF‐kB axis in paraventricular nucleus on ventricular arrhythmias induced by sympathetic hyperexcitation in post‐myocardial infarction rats

**DOI:** 10.1111/jcmm.17309

**Published:** 2022-04-08

**Authors:** Kang Wang, Shuling You, Hesheng Hu, Xiaolu Li, Jie Yin, Yugen Shi, Lei Qi, Pingjiang Li, Yuepeng Zhao, Suhua Yan

**Affiliations:** ^1^ Department of Cardiology Shandong Qianfoshan Hospital Cheeloo College of Medicine Shandong University Jinan Shandong China; ^2^ Adicon Clinical Laboratories.Inc. Department of Pathology Wangkai Infectious Diseases Hospital of Zaozhuang City Zaozhuang Shandong China; ^3^ Department of Cardiology Shandong Medicine and Health Key Laboratory of Cardiac Electrophysiology and Arrhythmia The First Affiliated Hospital of Shandong First Medical University & Shandong Provincial Qianfoshan Hospital Jinan Shandong China; ^4^ Department of Emergency Medicine Shandong Medicine and Health Key Laboratory of Emergency Medicine The First Affiliated Hospital of Shandong First Medical University & Shandong Provincial Qianfoshan Hospital Jinan Shandong China; ^5^ Shandong First Medical University & Shandong Academy of Medical Sciences Jinan China

**Keywords:** malignant ventricular arrhythmias, myocardial infarction, paraventricular nucleus, sympathetic hyperactivation, the toll‐like receptor 4/myeloid differentiation primary response 88/nuclear factor‐kappa B axis

## Abstract

Sympathetic activation after myocardial infarction (MI) leads to ventricular arrhythmias (VAs), which can result in sudden cardiac death (SCD). The toll‐like receptor 4 (TLR4)/myeloid differentiation primary response 88 (MyD88)/nuclear factor‐kappa B (NF‐kB) axis within the hypothalamic paraventricular nucleus (PVN), a cardiac‐neural sympathetic nerve centre, plays an important role in causing VAs. An MI rat model and a PVN‐TLR4 knockdown model were constructed. The levels of protein were detected by Western blotting and immunofluorescence, and localizations were visualized by multiple immunofluorescence staining. Central and peripheral sympathetic activation was visualized by immunohistochemistry for c‐fos protein, renal sympathetic nerve activity (RSNA) measurement, heart rate variability (HRV) analysis and norepinephrine (NE) level detection in serum and myocardial tissue measured by ELISA. The arrhythmia scores were measured by programmed electrical stimulation (PES), and cardiac function was detected by the pressure–volume loop (P‐V loop). The levels of TLR4 and MyD88 and the nuclear translocation of NF‐kB within the PVN were increased after MI, while sympathetic activation and arrhythmia scores were increased and cardiac function was decreased. However, inhibition of TLR4 significantly reversed these conditions. PVN‐mediated sympathetic activation via the TLR4/MyD88/NF‐kB axis ultimately leads to the development of VAs after MI.

## 1 INTRODUCTION

Ventricular arrhythmias (VAs) are the most frequent arrhythmia pattern after myocardial infarction (MI), and they are the main cause of eventual sudden cardiac death (SCD).^1^ Cardiac adrenergic activation and sympathetic excitation after MI are central factors in the development of VAs.^2^ Therefore, inhibiting sympathetic overactivation after MI is significantly important to reduce the incidence of VAs.

Neuroinflammation in the paraventricular nucleus (PVN) of the hypothalamus is a key factor in increased sympathetic activity, which is the basis for the pathogenesis of many cardiovascular diseases, including MI.^3^, ^4^ Numerous studies have shown that acute myocardial infarction (AMI) induces an early inflammatory response, resulting in a neuroinflammatory response and oxidative stress within the PVN, which leads to neuronal excitation within the PVN and the promotion of stellate ganglion (STG) remodelling and inflammatory infiltration, eventually leading to cardiac sympathetic excitation.^5^, ^6^, ^7^, ^8^ However, the exact mechanism of how PVN induces sympathetic excitation and ultimately leads to VAs in the heart is unclear.

Toll‐like receptor 4 (TLR4) plays a critical role in immune processes such as proinflammatory cytokine (PIC) secretion and microglia phagocytosis and is expressed on all major central nervous system (CNS) cells. TLR4‐specific antagonists can inhibit neuroinflammation by reducing the overproduction of inflammatory mediators.^6^, ^9^ It has been shown that the TLR4/myeloid differentiation primary response 88 (MyD88)/nuclear factor (NF‐kB) signalling pathway mediates neuroinflammation in CNS diseases such as Alzheimer's disease (AD), Parkinson's disease (PD), subarachnoid haemorrhage (SAM) and vascular dementia.^10^, ^11^, ^12^ It has also been shown that the TLR4/MyD88/NF‐kB signalling pathway plays a very important role in the inflammatory damage to cardiomyocytes caused by cardiovascular diseases such as heart failure (HF) after AMI, myocardial fibrosis (MF), hypoxia/reoxygenation (H/R) and coronary microembolism (CME).^13^, ^14^, ^15^, ^16^ A recent study claimed that the TLR4/MyD88/NF‐kB signalling pathway is upregulated in the rostral ventrolateral medulla (RVLM) of stress‐induced hypertensive rats (SIHRs), promoting a neuroinflammatory response in the RVLM that increases sympathetic activity and leads to increased blood pressure.^17^ Therefore, we hypothesized that the TLR4/MyD88/NF‐kB signalling pathway of the PVN also mediates peripheral sympathetic activity. We aimed to inhibit TLR4 in the PVN with adeno‐associated virus (AAV) with shTLR4 to investigate whether it could reduce the occurrence of VAs after MI by decreasing downstream MyD88 and inhibiting NF‐kB nuclear translocation and peripheral sympathetic nerve activity.

## 2 MATERIALS AND METHODS

### 2.1 Animals

Male Sprague‐Dawley (SD) rats weighing 240–280 g (Beijing Vital River Laboratory Animal Technology Co., Ltd.) were used in this study. All experimental procedures were performed following the guidelines of the Institute of Medical Ethics, Shandong University School of Medicine. Rats were placed in identical standard rat cages with 5 rats per cage under a 12‐h light–12‐h dark cycle at room temperature with free access to feed and drinking water.

### 2.2 Myocardial infarction modelling

Every rat was anaesthetized and tracheally intubated, and a left‐sided thoracic incision was made between the 3rd and 4th ribs to open the muscular layer and pericardium. The anterior descending branch of the left coronary artery (LAD) was then located between the pulmonary artery cone and the left auricle and ligated 1–2 mm below the left auricle.^18^ MI modelling criteria were that the rat ECG showed ST‐segment elevation, the myocardium turned grey below the ligated region, and there was reduced ventricular wall mobility. In the sham‐operated group, the procedure was the same as above, but the LAD was only threaded and not ligated. After thorax closure, the rats were placed on a warming pad at 37°C and placed in a cage after they had awakened.

### 2.3 Paraventricular nucleus microinjection

Every rat was anaesthetized and fixed on a brain stereotaxic apparatus (68002, RWD Life Science). The skull was exposed, and the instrument coordinates were zeroed at the point where the bregma comes into contact with the drill. Two holes of approximately 0.6 mm diameter were drilled into the rat skull according to the coordinates of X: ±0.4 mm, Y: −1.8 mm, avoiding damage to the brain during the operation. The location is shown in Figure S1 for methylene blue positioning. According to our previous study,^6^ we selected AAV with shTLR4 and its control virus (AAV with shCtrl) for the TLR4 knockdown and control groups, respectively, and both were injected into the PVN with a 0.5‐µl microinjector. The injection volume was 0.5 µl in all groups.

### 2.4 Experimental design

#### 2.4.1 Protocol 1

Surviving rats after the MI surgery were divided into 5 groups according to whether the LAD was ligated or not and the time that the rats were sacrificed: (1) sham, sham‐operated group (*n* = 8); (2) 1d, 1 day after MI (*n* = 8); (3) 3d, 3 days after MI (*n* = 7); (4) 5d, 5 days after MI (*n* = 5); and (5) 7d, 7 days after MI (*n* = 7).

#### 2.4.2 Protocol 2

Four weeks after PVN microinjection with AAV with shTLR4 or shCtrl, the rats underwent MI and were executed 3 days later. The surviving rats were divided into 4 groups according to the injected virus and whether the LAD was ligated during the infarction surgery: (1) sham + shCtrl (*n* = 15); (2) sham + shTLR4 (*n* = 14); (3) MI + shCtrl (*n* = 16); and (4) MI + shTLR4 (*n* = 15).

### 2.5 Heart rate variability measurement

Rats were connected to an ECG machine (lead II), recordings were obtained for 30 min, and heart rate variability (HRV) was analysed by LabChart Pro software (AD Instruments). High frequency (HF; 0.75–2.5 Hz) indicates parasympathetic tone, while low frequency (LF; 0.05–0.75 Hz) indicates parasympathetic and sympathetic tone. An increased LF/HF ratio indicates cardiac sympathetic imbalance.^6^, ^19^

### 2.6 Renal sympathetic nerve activity (RSNA) measurement

The left renal sympathetic nerve of rats was carefully isolated behind the peritoneum, cut distally and placed on top of a pair of platinum electrodes connected to a power lab (AD Instrument) to amplify, integrate and record the RSNA signal.

### 2.7 Pressure–volume (P‐V) loop study

A P‐V ring catheter was inserted into the left ventricle to record the pressure and volume of the left ventricle after the rats were anaesthetized. Data from all loops were formally recorded for 20 min for data analysis after a stable P‐V loop signal was obtained. The systolic blood pressure (SBP), diastolic blood pressure (DBP), mean arterial pressure (MAP), left ventricular end‐systolic pressure (LVESP), left ventricular end‐diastolic pressure (LVEDP), maximum slope of the left ventricular systolic pressure increment (dp/dtmax), maximum diastolic decremental pressure (dp/dtmin), ejection fraction (EF) and heart rate (HR) were calculated with LabChart Pro software (AD Instruments).

### 2.8 Programmed electrical stimulation (PES)

Electrodes were placed closely against the epicardial surface of the left ventricle, and signals were recorded using an animal biofunctional experiment system (LEAD‐7000; JJET). Electrical stimulation was programmed using a cycle length of 120 ms with 8 pacing (S0) followed by single (S1), two (S2) and three (S3) additional stimulation steps.^20^ Stimulation was interrupted in the presence of myocardial effective refractory period or VAs. Arrhythmia scoring criteria: 0 points for no VAs or <10 premature ventricular beat, PVB; 1 point for ≥10 PVB; 2 points for only 1 ventricular tachycardia, VT (<120 s); 3 points for 1 VT (≥120 s) or multiple VT (<120 s); 4 points for multiple cumulative VT (>120 s); 5 points for ventricular fibrillation, VF; and 6 points for VF (lasting > 5 min) or death during observation.^21^

### 2.9 Collection of samples

Blood was collected from rats under deep anaesthesia, followed by centrifugating at 4°C for 20 min at 1000x *g*, and serum was collected. After perfused with saline and 4% Paraformaldehyde Fix Solution (Servicebio, G1101), the rats were executed and brain and heart tissues were collected. The brain tissues were divided into three groups: (1) PVN was carefully removed directly from both sides of the third ventricle; (2) brain tissue was frozen after being covered with OCT embedding agent (Servicebio, G6059); and (3) brain tissue was embedded in paraffin. The heart tissues were divided into two groups: (1) 3 mm of myocardial tissue surrounding the infarct region of the left ventricle in rats was carefully removed, and (2) the whole heart tissue was embedded in paraffin.

### 2.10 Western blot

Proteins were extracted from the PVN samples by protein lysates, and the protein concentrations were measured using the BSA assay. Equal amounts of protein samples were separated by a 10%–12.5% gel and incubated with TLR4 antibody (GeneTex, GTX64330, 1:1000), anti‐MyD88 antibody (Abcam, ab219413, 1:1000), anti‐NF‐kB p65 (Abcam, ab16502, 1:1000), anti‐IkB alpha antibody (Abcam, ab32518, 1:1000), GAPDH (Cell Signaling, #2118, 1:1000) and anti‐histone antibody (Bios, bs‐17422R, 1:1000) overnight at 4°C. Then, they were incubated with HRP goat anti‐mouse IgG (H + L) (ABclonal, AS003, 1:2000) and goat anti‐rabbit IgG‐HRP (Absin Bioscience Inc., abs20040ss, 1:5000) for 1 h at room temperature on a shaker. The protein strips were developed with Stain‐Free Western blotting technology (Bio‐Rad) and analysed with ImageJ software (Bio‐Rad, v.1.8.0.112).

### 2.11 Multiplex immunofluorescence staining

Frozen sections of brain tissue were coincubated with primary antibody staining for 2 h, with secondary antibody for 1 h and then with signal amplification solution (Absin Bioscience Inc., abs50012) for 10 min. The sections were covered with DAPI (Aqueous, Fluoroshield, Abcam, ab104139). Fluorescence images were viewed and captured under a confocal microscope (Nikon, A1 HD25/A1R HD25) and analysed with NIS‐Elements Viewer (Nikon, v. 5.21.00). The staining order and correspondence are shown in Table 1.

**TABLE 1:** Multiplex immunofluorescence staining table

Staining order	1	2	3
Primary antibody	Anti‐TLR4 antibody (GeneTex, GTX64330, 1:100)	Anti‐MyD88 antibody (Abcam, ab133739, 1:500)	Anti‐NF‐kB p65 (Abcam, ab16502, 1:500)
Secondary antibody	Alexa Fluor 594‐conjugated AffiniPure goat anti‐rabbit IgG (Jackson ImmunoResearch Laboratories, Inc., 147909, 1:200)		
Fluorescent dyes	TSA−570	TSA−520	TSA−650
Wavelength	555	488	647
colour observed under a confocal microscope	Red	Green	Black (Changed to cyan with PS to make the it clearer)

### 2.12 Immunohistochemistry

Brain tissue was collected, embedded in paraffin and cut into 5‐µm sections along the coronal section. The sections were incubated with c‐Fos antibody (Santa Cruz Biotechnology, Inc., sc‐166940, 1:100) at 4°C overnight and incubated with goat anti‐mouse (HRP) antibody (Servicebio, G1214, 1:200) for 1 h at room temperature. The sections were then stained with a DAB Chromogenic Kit (Servicebio, G1212) and counterstained with haematoxylin. Positive neurons (brown) of three consecutive sections of the PVN on both sides of each rat were observed under the microscope and counted manually, and the mean was recorded for subsequent analysis.

### 2.13 Masson staining

Heart tissue was collected along the cross‐section of the heart between the upper and lower edges of the left ventricular infarct zone, embedded in paraffin, cut into sections of 5‐mm thickness, stained with the Masson's Trichrome Stain Kit (G1340, Solarbio) according to the instructions and observed under a microscope (Olympus Corporation, SZX10). The infarct size (%) = 1/2*(epicardial circumference + endocardial circumference)/left ventricular circumference*100%.^22^

### 2.14 Measurement of norepinephrine (NE) levels in plasma and myocardial tissue

Plasma and myocardial tissue levels of NE were measured using NA/NE (Noradrenaline/Norepinephrine) ELISA Kits (Elabscience, E‐EL‐0047c) according to the instructions. Finally, the optical density (OD) values were measured by using a Flexstation3 multifunctional microplate reader (Molecular Devices).

### 2.15 Statistical analyses

GraphPad Prism (GraphPad Software) was used for statistical analysis. The one‐way analysis of variance (ANOVA) followed by Tukey's test was used for multiple comparisons to compare differences between groups. The unpaired *t*‐test was used to compare difference between two groups. Significant differences were indicated as *p* < 0.05. *, *p* ≤ 0.05; **, *p* ≤ 0.01; and ***, *p* ≤ 0.001. Values for statistical plots are expressed as the mean ± standard deviation (SD).

## 3 RESULTS

### 3.1 Increased TLR4/MyD88/NF‐kB in the PVN after MI

**FIGURE 1:**
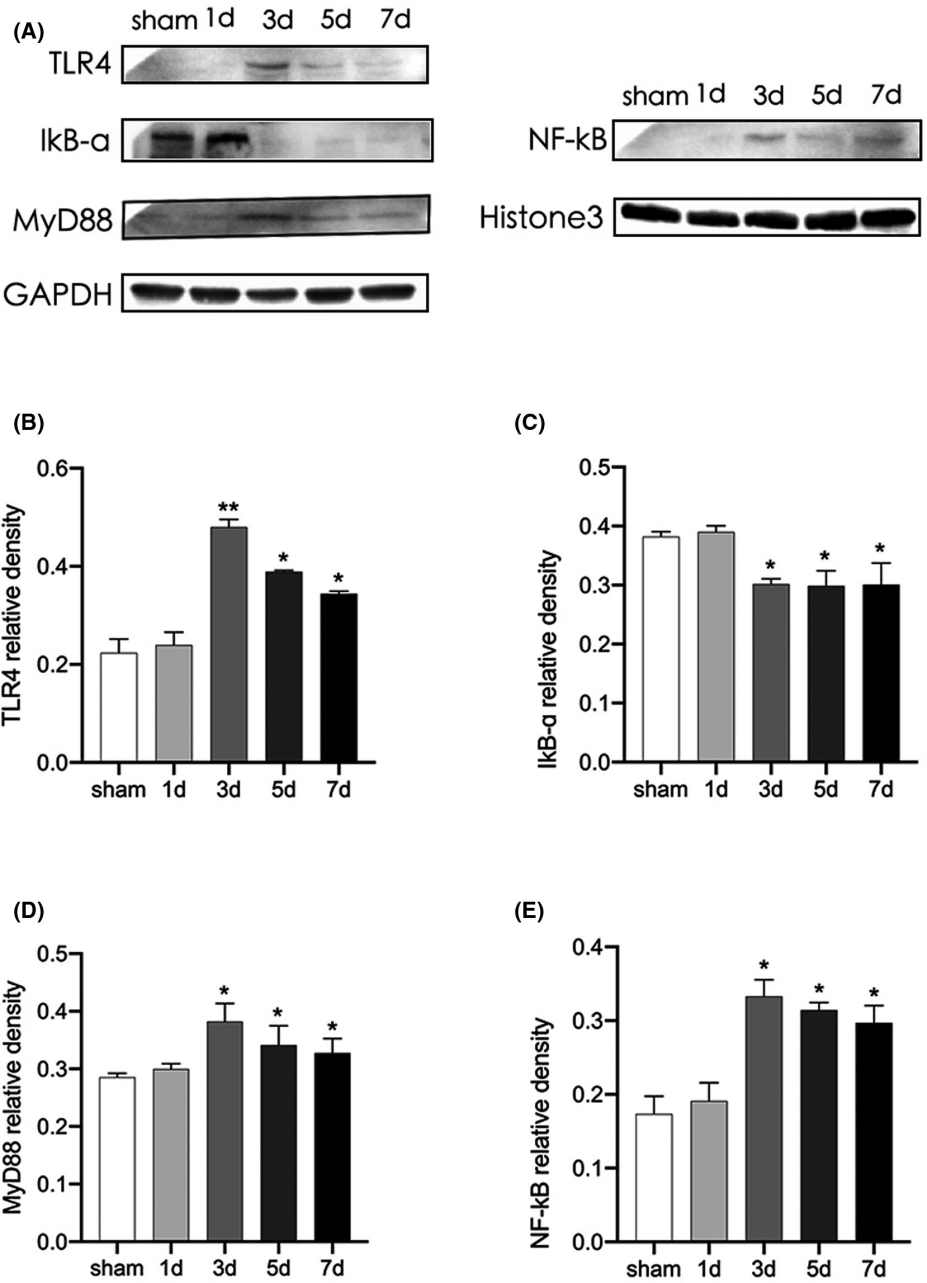
Protein expression in the paraventricular nucleus (PVN) was detected by Western blotting. (A) is a representative image of the sham, MI1d, MI3d, MI5d and MI7d groups. Normalized treatment: TLR4, IkB‐a, MyD88 compared with GAPDH (B‐D) and NF‐kB compared with Histone 3 (E). Values are the mean ± SD. Sham, *n* = 8; 1d, *n* = 8; 3d, *n* = 7; 5d, *n* = 5; 7d, *n* = 7. *p < 0.05, **p < 0.01 compared with sham group. Abbreviations: 1d, 1 day after MI; 3d, 3 days after MI; 5d, 5 days after MI; 7d, 7 days after MI. MI, myocardial infarction

The PVNs of the sham group and rats sacrificed on Days 1, 3, 5 and 7 after MI were examined by Western blotting (Figure 1A). TLR4, MyD88 and NF‐kB increased on Day 1 after MI, although there were no statistically significant differences. The expression levels of the three molecules increased in parallel, reaching the highest level on Day 3, and were significantly higher than those of the sham group until Day 7 after MI. IkB‐a also dropped the most on the third day (Figure 1B‐E).

### 3.2 Inhibition of TLR4 within the PVN suppressed MyD88/NF‐kB levels

**FIGURE 2:**
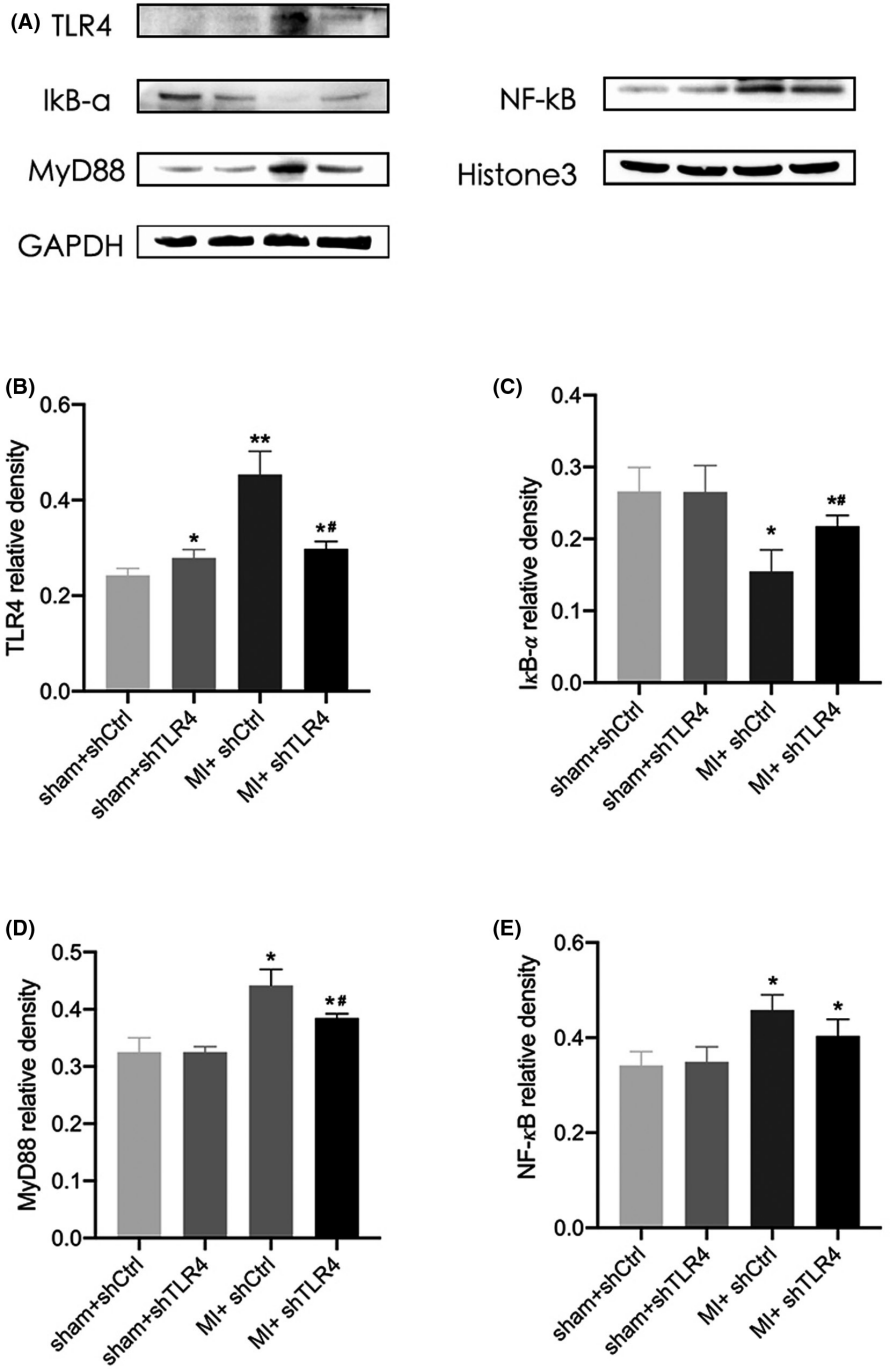
Protein expression in the paraventricular nucleus (PVN) was detected by Western blotting. (A) is a representative picture of the protein bands of the PVN from the four groups, namely sham + shCtrl, sham + shTLR4, MI + shCtrl and MI + shTLR4. Normalization: TLR4, IkB‐a, MyD88 compared with GAPDH (B‐D) and NF‐kB compared with Histone 3 (E). Values are the mean ± SD. sham + shCtrl, *n* = 5; sham + shTLR4, *n* = 4; MI+shCtrl, *n* = 6; MI + shTLR4, *n* = 5. *p < 0.05, **p < 0.01 compared with sham group. ^#^ *p* < 0.05 compared with MI + shCtrl. Abbreviations: MI, myocardial infarction; shCtrl, control virus; shTLR4, TLR4‐inhibiting virus

**FIGURE 3:**
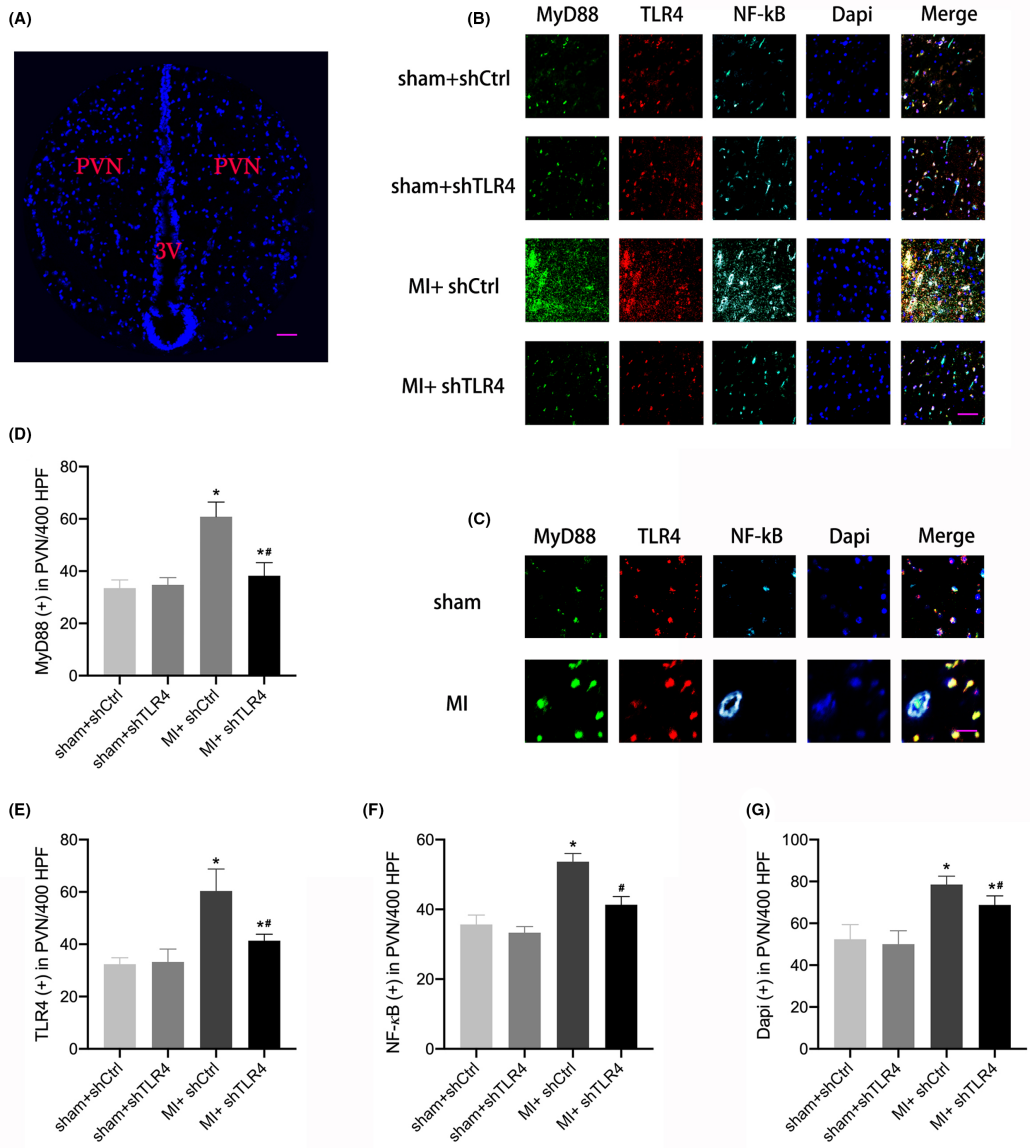
(A) is the representative immunofluorescence image of paraventricular nucleus (PVN) in a rat, marking the location of PVN on both sides of the third ventricle (400 HPF), bar = 30 µm. The blue dots represented the nucleus. (B) is representative image of multiplex immunofluorescence staining from the sham + shCtrl, sham + shTLR4, MI + shCtrl and MI + shTLR4 groups of TLR4, MyD88 and NF‐kB (400 HPF) in the PVN, bar = 30 µm. (C) is representative image of multiplex immunofluorescence staining from the sham and MI groups of TLR4, MyD88 and NF‐kB (400 HPF) in the PVN, bar = 15 µm. The number of TLR4‐, MyD88‐, NF‐kB‐ and nuclei‐positive cells in each group was counted and is presented as the mean ± SD (D‐G). *n* = 5 in each group. *p < 0.05 compared with sham group. ^#^ *p* < 0.05 compared with MI + shCtrl. Abbreviations: MI, myocardial infarction; PVN, paraventricular nucleus; shCtrl, control virus; shTLR4, TLR4‐inhibiting virus; 3V, the third ventricle

Adeno‐associated virus‐shTLR4 was injected into the PVN of rats as described in the methods of this study, and Figure S2 shows the expression and distribution of GFP within the PVN, which demonstrated that the PVN was effectively transfected by adenovirus. Western blot analysis showed that the TLR4 and MyD88 levels and cytosolic NF‐kB increased after MI, and the level of IkB‐a was reduced, whereas this change was significantly reduced in the MI + shTLR4 group (Figure 2). We located PVN on both sides of the rat third ventricle by localizing it (Figure 3A), and subsequently observed and recorded each molecule by computer. The multiplex immunofluorescence results also showed increased levels of TLR4, MyD88 and the number of nuclei in MI + shCtrl group, while the MI + shTLR4 group exhibited inhibition of this change (Figure 3B,D‐G). We further enlarged the fluorescence image of sham group and MI group to better observe nuclear translocation of NF‐kB (Figure 3C). Masson staining showed a significantly larger heart area in the MI group than in the sham group. However, injection of shTLR4 did not have a significant effect on the MI infarct area in rats (Figure S3).

### 3.3 Inhibition of TLR4 inhibited sympathetic activation after MI

**FIGURE 4:**
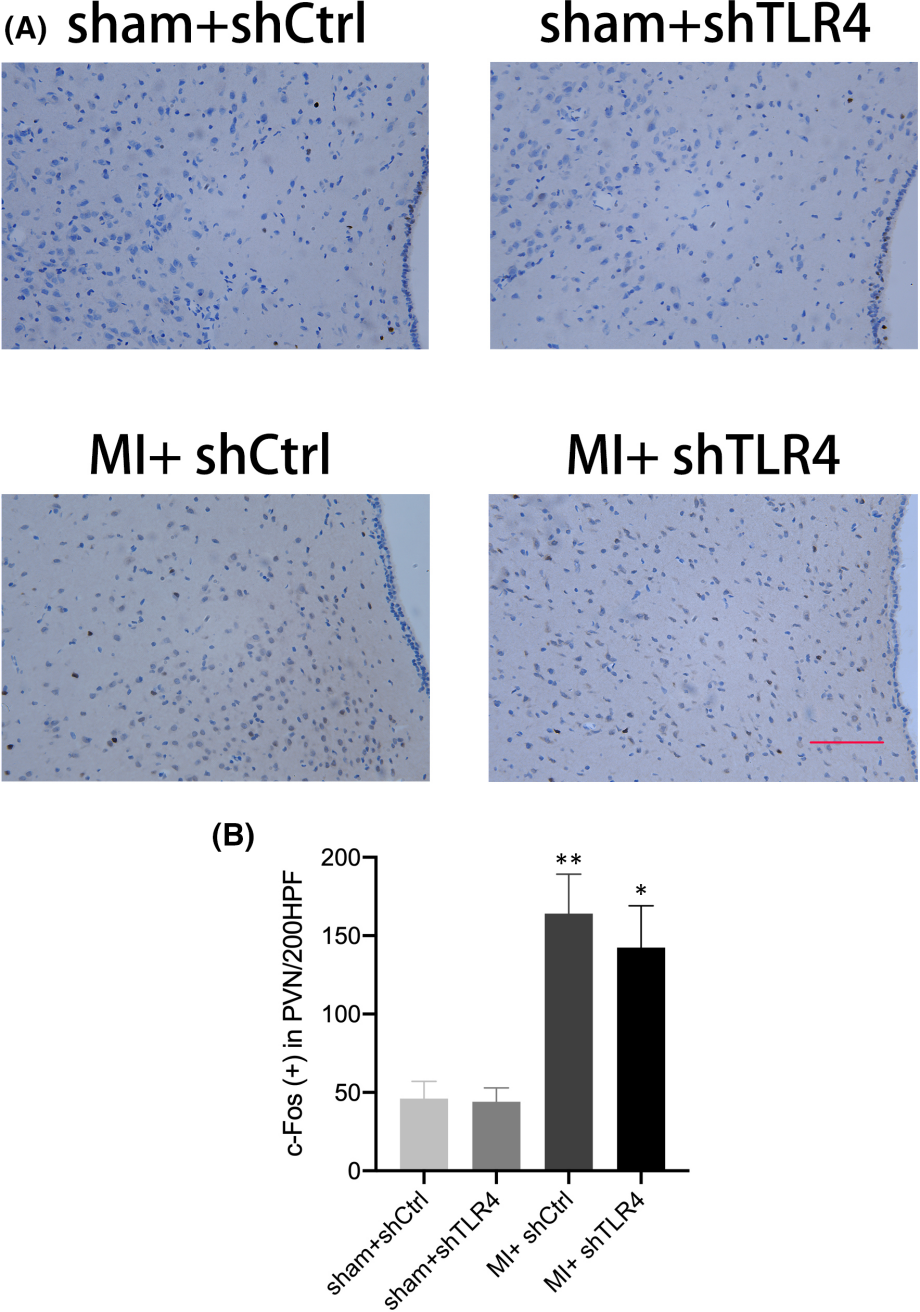
(A) is a representative image of immunohistochemical staining for c‐fos protein in the PVN, the amount of which reflects the degree of neuronal activation. The fos protein was stained brown, and the nucleus was stained blue (200 HPF), bar = 50 µm. The statistical plots of the fos protein levels in the four groups, sham + shCtrl, sham + shTLR4, MI + shCtrl and MI + shTLR4, are presented as the mean ± SD (B). *n* = 5 in each group. *p < 0.05 compared with sham group. Abbreviations: MI, myocardial infarction; shCtrl, control virus; shTLR4, TLR4‐inhibiting virus

**FIGURE 5:**
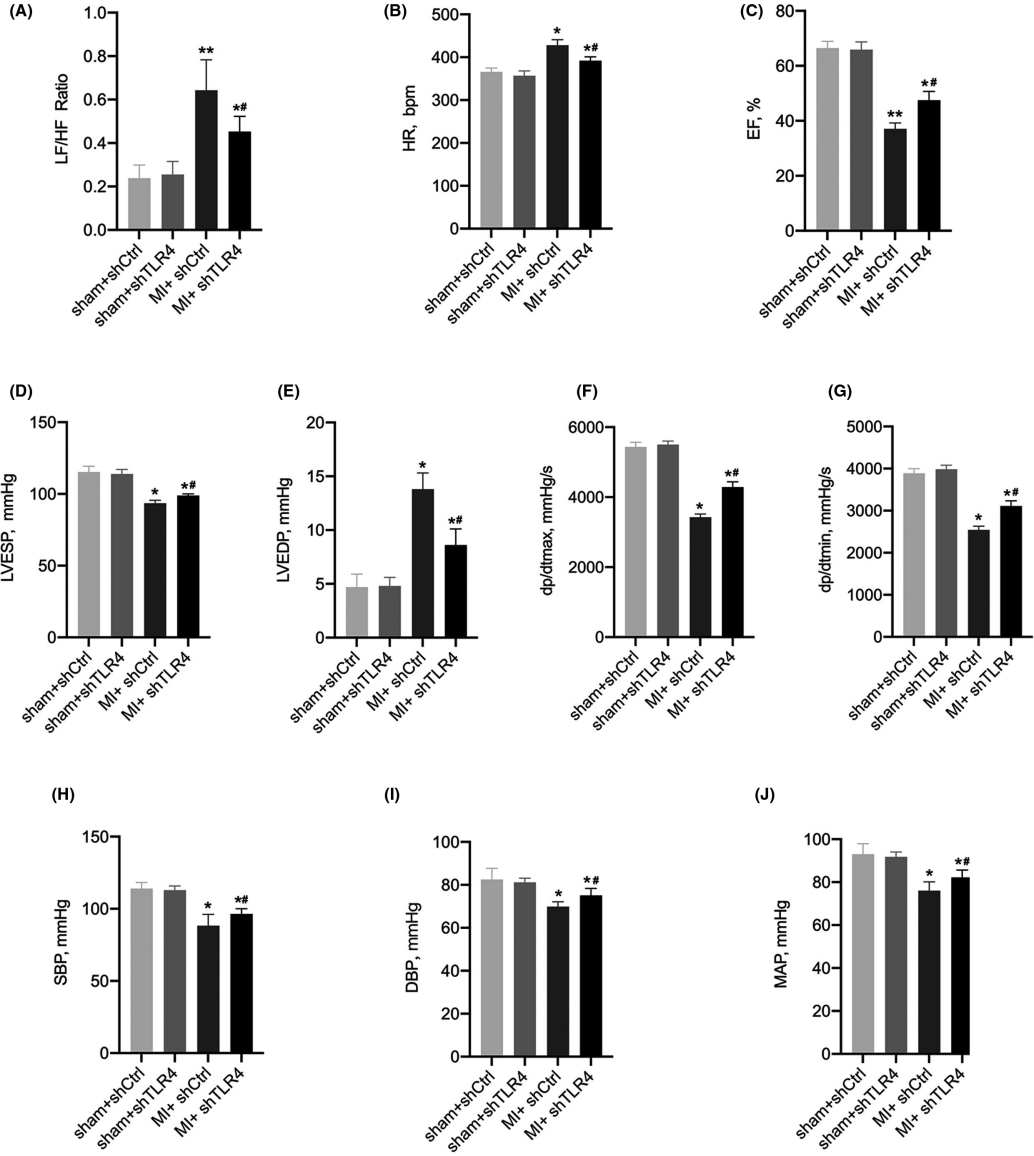
(A and B) are statistical plots of the HRV analysis for each group of rats, and (C‐J) represent the cardiac function of each group of rats. The four groups of rats were the sham + shCtrl (*n* = 15), sham + shTLR4 (*n* = 14), MI + shCtrl (*n* = 16) and MI + shTLR4 (*n* = 15). Values are the mean ± SD. *p < 0.05, **p < 0.01 compared with sham group. ^#^ *p* < 0.05 compared with MI + shCtrl. Abbreviations: DBP, diastolic blood pressure; dp/dtmax, maximum slope of the left ventricular systolic pressure increment; dp/dtmin, maximum diastolic decremental pressure; EF, ejection fraction; HF, high frequency; HR, heart rate; LF, low frequency; LVESP, left ventricular end‐systolic pressure; LVEDP, left ventricular end‐diastolic pressure; MAP, mean arterial pressure; MI, myocardial infarction; SBP, systolic blood pressure; shCtrl, control virus; shTLR4, TLR4‐inhibiting virus

**FIGURE 6:**
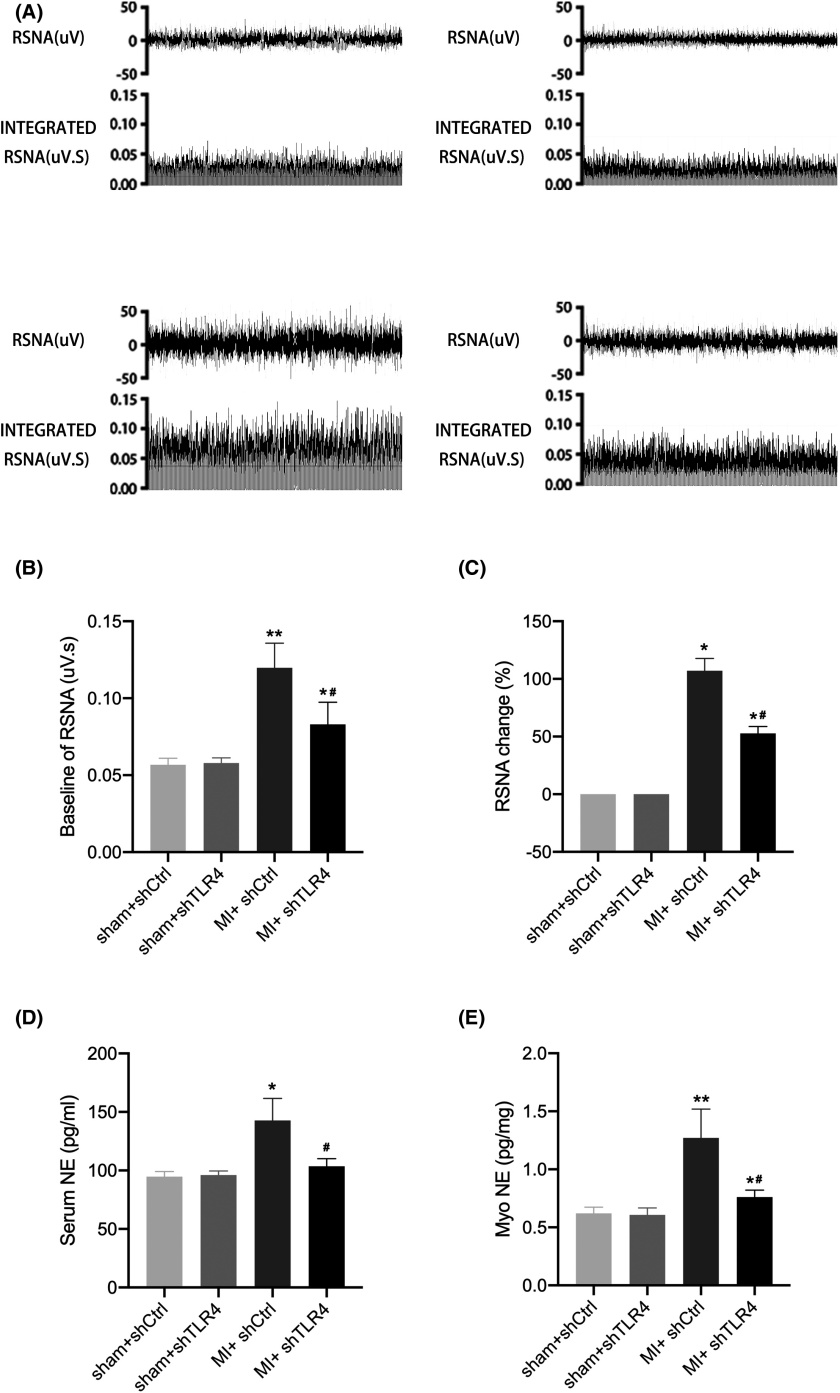
(A) is a representative picture of RSNA of sham + shCtrl, sham + shTLR4, MI + shCtrl and MI + shTLR4 rats. (B) is the baseline RSNA, and (C) is the change in RSNA compared to that of the sham + shCtrl group. NE levels in serum (D) and myocardial tissue (E) as detected by ELISA. Values are the mean ± SD. sham + shCtrl, *n* = 15; sham + shTLR4, *n* = 14; MI + shCtrl, *n* = 16; MI + shTLR4, *n* = 15. *p < 0.05, **p < 0.01 compared with sham group. ^#^ *p* < 0.05 compared with MI + shCtrl. Abbreviations: MI, myocardial infarction; NE, norepinephrine; RSNA, renal sympathetic nerve activity; shCtrl, control virus; shTLR4, TLR4‐inhibiting virus

In the MI group and the sham group, the amount of c‐fos protein in the PVN region that was stained brown indicated the degree of central sympathetic activation (Figure 4A). A comparison of the statistical results of the MI + shTLR4 group with the MI + shCtrl group showed that central sympathetic activation after MI was significantly inhibited after reducing the level of TLR4 within the PVN (Figure 4B). Inhibition of TLR4 within the PVN resulted in a significantly higher HRV and LF/HF ratio in the MI + shCtrl group than in the sham + shCtrl group, whereas MI + shTLR4 reduced this change (Figure 5A,B). NE levels varied similarly to RSNA in each group, while RSNA and NE represented the degree of peripheral sympathetic activation. Thus, the results showed that peripheral sympathetic activation after MI was effectively reversed by inhibiting TLR4 (Figure 6).

### 3.4 Inhibition of TLR4 reduced the incidence of VAs and improved cardiac function

**FIGURE 7:**
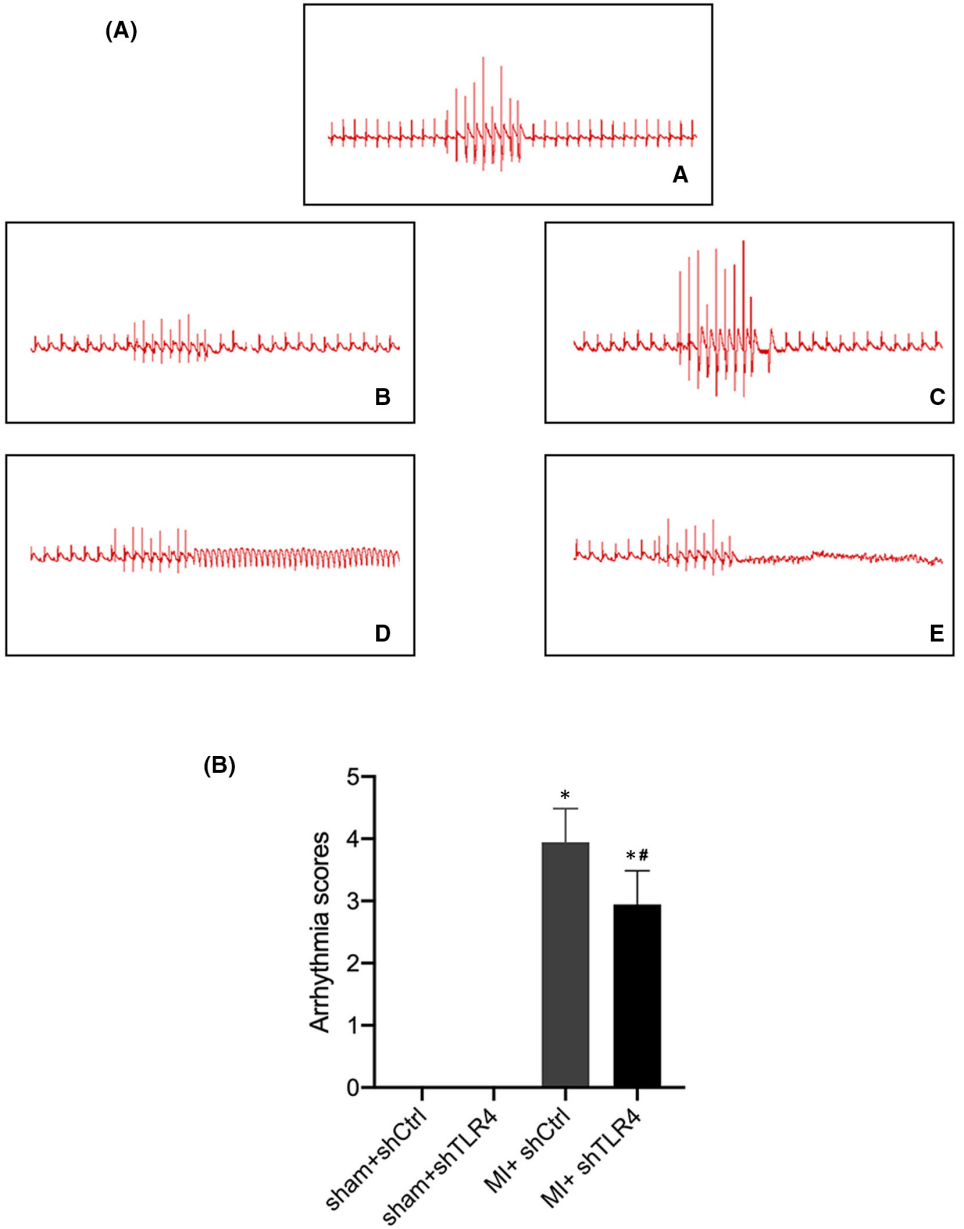
Typical ECG of rats with PES including rats in the sham‐operated group with unstimulated arrhythmias (A). (a) and rats in the MI group with unstimulated arrhythmias (b), stimulated ventricular premature beats (c), ventricular tachycardia (d) and ventricular fibrillation (e). (B) is the mean ± SD of the ventricular arrhythmia scores in sham + shCtrl (*n* = 15), sham + shTLR4 (*n* = 14), MI + shCtrl (*n* = 16) and MI + shTLR4 (*n* = 15) groups. *p < 0.05 compared with sham group. #*p* < 0.05 compared with MI + shCtrl. Abbreviations: MI, myocardial infarction; shCtrl, control virus; shTLR4, TLR4‐inhibiting virus

Typical images of VAs induced by PES are shown in Figure 7A. It is clear that the inhibition of TLR4 within the PVN significantly reduced the incidence of VAs after MI according to the significant differences in the arrhythmia scores (Figure 7B). Additionally, inhibition of TLR4 within the PVN was able to improve post‐MI cardiac function (Figure 5C‐J).

## 4 DISCUSSION

Studies have shown that many cardiovascular diseases, such as MI, HF and hypertension, are characterized by reduced cardiac function due to peripheral inflammation associated with increased central sympathetic tone, while neuroinflammation of the PVN in turn leads to increased central sympathetic tone.^4^ Under hypertensive conditions, the PVN activates and releases NE,^23^ an excitatory neurotransmitter, thereby promoting sympathetic output.^24^, ^25^ In studying diseases such as memory impairment and neurological damage after cerebral ischaemia‐reperfusion, animal models of cerebral ischaemic stroke reflect that the TLR4/MyD88/NF‐kB axis is involved in neuroinflammation.^26^, ^27^ We performed a study on the role of TLR4 in the mechanism of PVN‐mediated sympathetic activation after MI. Treatment with shTLR4 adenovirus, which inhibited the levels of MyD88 and NF‐kB, was able to suppress central and peripheral sympathetic activation, attenuate the incidence of malignant VAs and improve cardiac function.

Studies have shown that peripheral proinflammatory cytokines such as TNF‐a are released after MI, leading to blood–brain barrier dysfunction in the PVN and leakage from the brain endothelium, thereby inducing neuroinflammation^28^ and activating sympathetic nerves throughout the body.^29^ Microglia are innate immune cells of the central nervous system (CNS). Microglia, activated after MI, mediate the production and release of proinflammatory cytokines through TLR4 locating on the cell membrane and its downstream molecules, causing neuroinflammation in the CNS,^30^ thereby causing sympathetic excitation.^8^ Inhibition of TLR4 could inhibit neuroinflammation and thus inhibit sympathetic overactivation. Our previous study also supported that the activated TLR4 and microglia shared a common localization.^6^ The level of TLR4 in the PVN was significantly increased in rats with MI, and the level of Iba‐1 was also increased, which means that the PVN was activated; inhibition of TLR4 in the PVN could inhibit sympathetic activation. The above data provide evidence for a potential role of TLR4 within the PVN in the development of VAs following MI. We further explored the role of TLR4 in cardiac function. Our study shows that inhibition of TLR4 in the PVN reduces the incidence of VAs and improves cardiac function after MI. Ultimately, AAV‐shTLR4 inhibits sympathetic activation after MI by suppressing the neuroinflammatory response within the PVN. We subsequently investigated the downstream MyD88/NF‐kB axis in depth.

Activation of TLR4 on the microglial cell membrane of the PVN leads to the activation of IkB kinase (IKK) in a MyD88‐dependent manner, thereby allowing IkB‐a, which locks NF‐kB in the cytoplasm when the cell is at rest, to be phosphorylated and degraded, ultimately leading to the NF‐kB complex (p50/p65) being phosphorylated and entering the nucleus.^31^, ^32^ TLR4 is highly expressed in microglia.^33^ Pinocembrin is a TLR4 inhibitor that has been approved by the Chinese Food and Drug Administration (CFDA) as a therapeutic agent for ischaemic stroke and is currently in phase II clinical trials. Pinocembrin's pharmacological therapeutic potential is primarily neuroprotective as it maintains the integrity of the blood‐brain barrier and has anti‐inflammatory effects by inhibiting the production of proinflammatory cytokines such as TNF‐a, IL‐1b and IL‐6; this action suppresses the level of MyD88 downstream of TLR4 and inhibits NO production in the NF‐kB pathway.^34^, ^35^ Our AAV‐shTLR4 acts in the same way as pinocembrin. In our study, activated TLR4 colocalized with the microglial marker Iba‐1,^6^ while TLR4, MyD88 and NF‐kB also colocalized. Inhibition of TLR4 was able to significantly inhibit the level of MyD88 and the nuclear translocation of NF‐kB. Therefore, we hypothesized that TLR4/MyD88/NF‐kB was activated in the microglia of the PVN after MI, resulting in a neuroimmune inflammatory response, which caused central and peripheral sympathetic excitation and ultimately led to the development of malignant VAs.

Although our results demonstrate increased levels of TLR4 and MyD88 within the PVN following MI, as well as increased NF‐kB nuclear translocation. The increases in all three of them were suppressed after the inhibition of TLR4, along with suppressed sympathetic activation and improved cardiac function. However, we cannot exclude that TLR4 does not act via the MyD88/NF‐kB pathway but acts directly on microglia. Current research on the role of the TLR4/MyD88/NF‐kB axis in the PVN in cardiovascular disease has mainly focused on hypertension.^36^, ^37^, ^38^ In future studies, we will continue to investigate whether TLR4 directly promotes inflammatory responses in microglia via the MyD88/NF‐kB pathway and the mechanism of this signalling axis on VAs leading to SCD after MI.

## CONFLICT OF INTEREST

The authors declare that there is no conflict of interest.

## AUTHOR CONTRIBUTIONS

**Suhua Yan:** Conceptualization (equal); Methodology (equal); Software (equal). **Kang Wang:** Data curation (equal); Writing – original draft (equal). **Shuling You:** Investigation (equal); Visualization (equal). **Hesheng Hu:** Supervision (equal). **Xiaolu Li:** Software (equal); Validation (equal). **Jie Yin:** Writing – review & editing (equal). **Yugen Shi:** Supervision (equal). **Lei Qi:** Software (equal); Validation (equal). **Pingjiang Li:** Writing – review & editing (equal). **Yuepeng Zhao:** Conceptualization (equal); Data curation (equal); Formal analysis (equal).

## Supporting information

Figure S1

Figure S2

Figure S3
