# Supplementary figures and images for: Effect of TLR4/MyD88/NF‐kB axis in paraventricular nucleus on ventricular arrhythmias induced by sympathetic hyperexcitation in post‐myocardial infarction rats
Source: J Cell Mol Med. 2022 Apr 8;26(10):2959–71. doi: 10.1111/jcmm.17309 (PMC9097841; doi:10.1111/jcmm.17309)

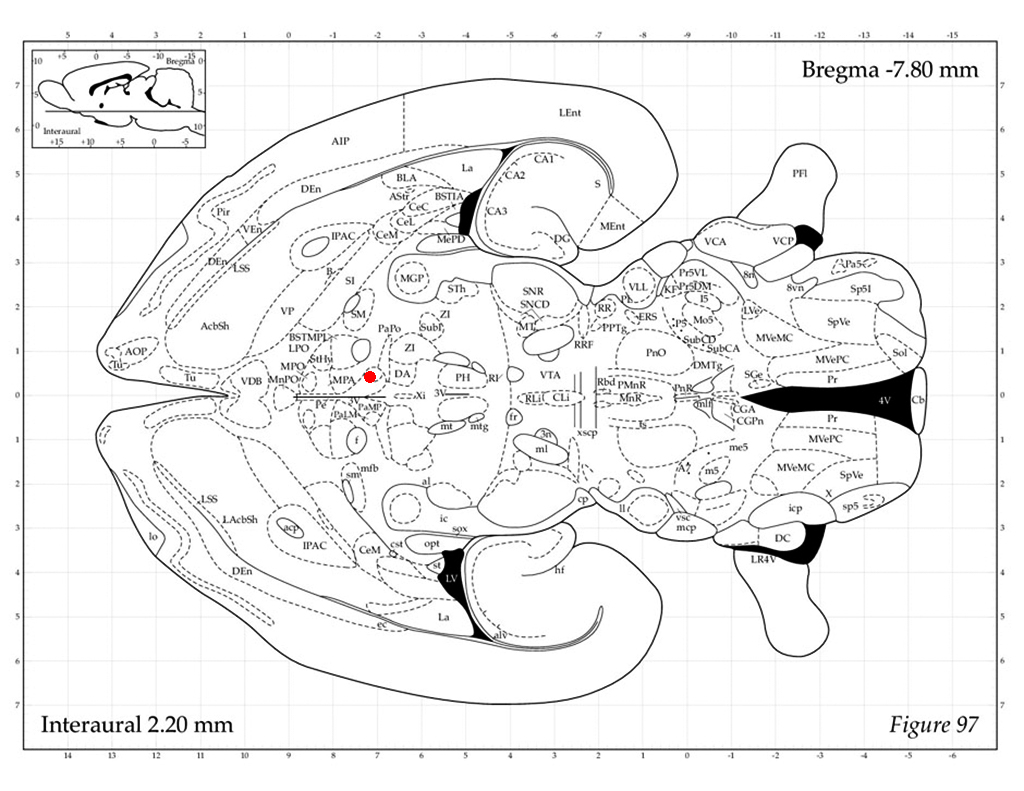

Supplement: Supplementary file 1 — Figure S1 [file JCMM-26-2959-s002.jpg]

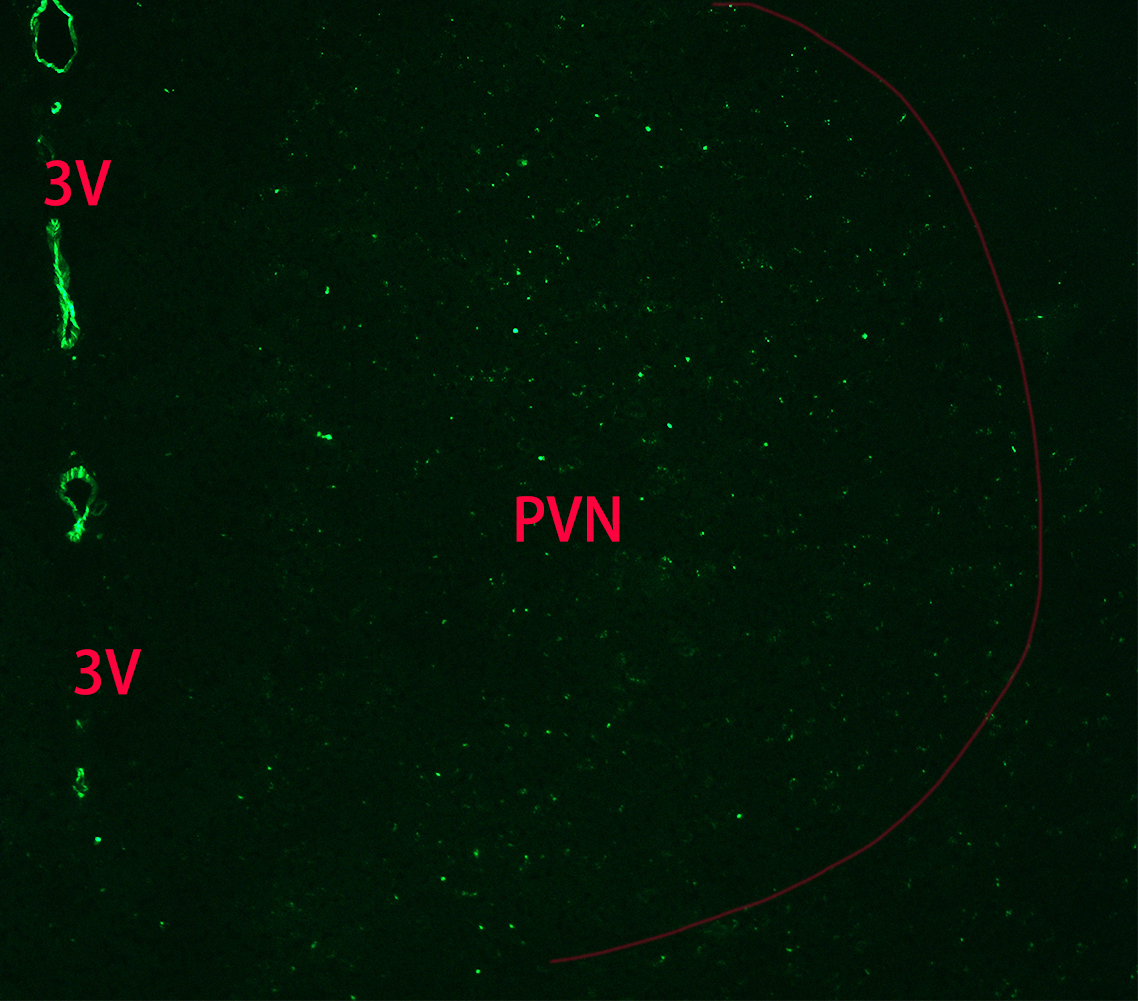

Supplement: Supplementary file 2 — Figure S2 [file JCMM-26-2959-s001.jpg]

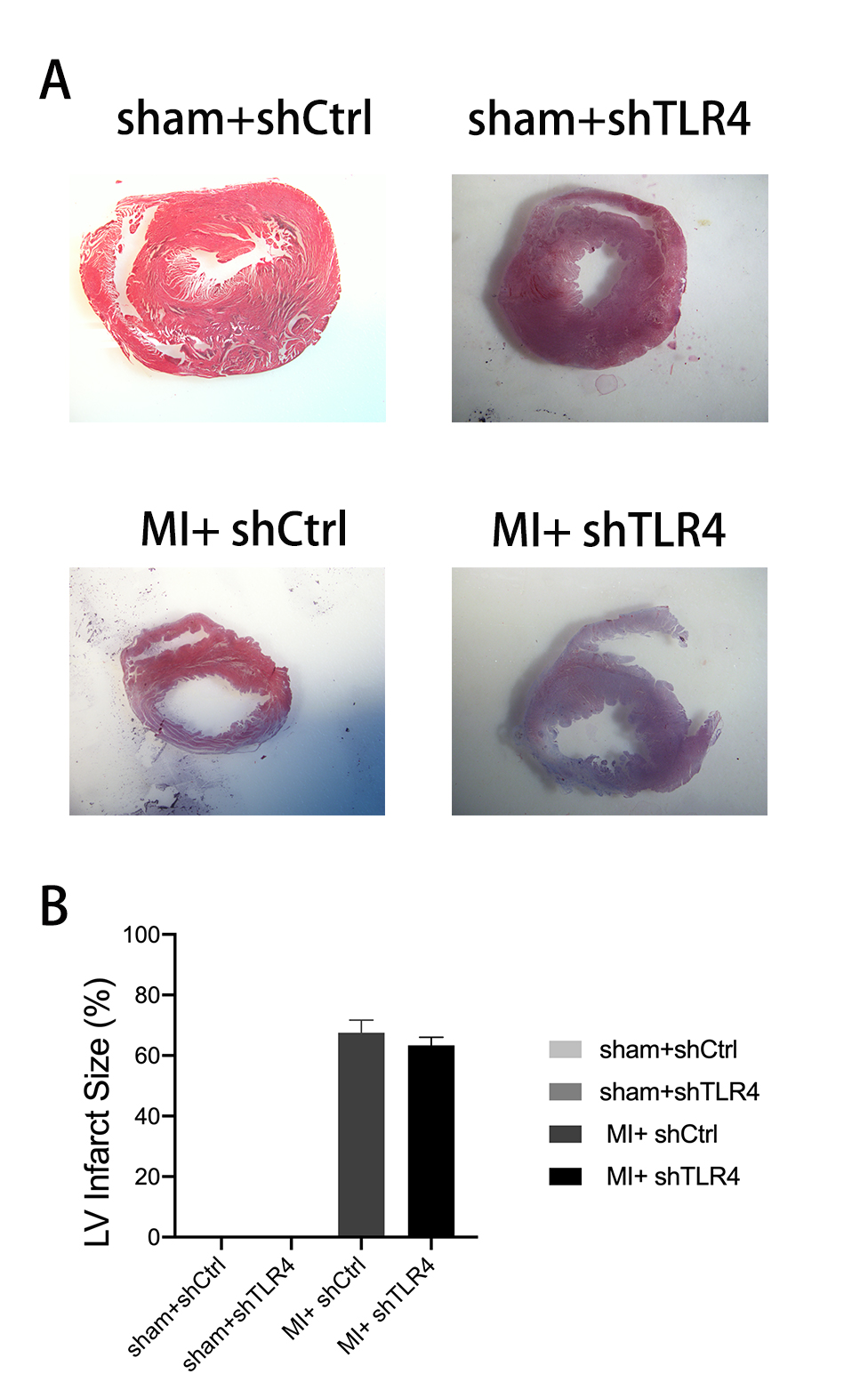

Supplement: Supplementary file 3 — Figure S3 [file JCMM-26-2959-s003.jpg]
